# Transfer Learning for Modeling Plasmonic Nanowire Waveguides

**DOI:** 10.3390/nano12203624

**Published:** 2022-10-16

**Authors:** Aoning Luo, Yuanjia Feng, Chunyan Zhu, Yipei Wang, Xiaoqin Wu

**Affiliations:** Key Laboratory of Optoelectronic Technology and Systems (Ministry of Education), College of Optoelectronic Engineering, Chongqing University, Chongqing 400044, China

**Keywords:** deep learning, transfer learning, plasmonics, nanowires, waveguides

## Abstract

Retrieving waveguiding properties of plasmonic metal nanowires (MNWs) through numerical simulations is time- and computational-resource-consuming, especially for those with abrupt geometric features and broken symmetries. Deep learning provides an alternative approach but is challenging to use due to inadequate generalization performance and the requirement of large sets of training data. Here, we overcome these constraints by proposing a transfer learning approach for modeling MNWs under the guidance of physics. We show that the basic knowledge of plasmon modes can first be learned from free-standing circular MNWs with computationally inexpensive data, and then reused to significantly improve performance in predicting waveguiding properties of MNWs with various complex configurations, enabling much smaller errors (~23–61% reduction), less trainable parameters (~42% reduction), and smaller sets of training data (~50–80% reduction) than direct learning. Compared to numerical simulations, our model reduces the computational time by five orders of magnitude. Compared to other non-deep learning methods, such as the circular-area-equivalence approach and the diagonal-circle approximation, our approach enables not only much higher accuracies, but also more comprehensive characterizations, offering an effective and efficient framework to investigate MNWs that may greatly facilitate the design of polaritonic components and devices.

## 1. Introduction

As important building blocks for next-generation nanophotonic components and devices, metal nanowires (MNW) are able to guide surface plasmon polaritons (SPPs) with a tight confinement in transverse cross-sections, providing a promising platform to manipulate light and light–matter interactions at the deep-subwavelength scale [1,2,3]. With the development of fabrication techniques, MNWs can be readily prepared via both top-down and bottom-up approaches, offering different geometric sizes and cross-sectional shapes (e.g., square [4] and pentagonal cross-sections [5] through electron-beam lithography and chemical synthesis, respectively) with intriguing waveguiding properties [6] in the visible and near-infrared regime for various applications, including all-optical light routing [7,8], ultrasensitive sensing [9,10], and plasmon lasing [11,12].

Retrieving waveguiding properties lies at the heart of the investigation of these MNWs, not only in uncovering relationships between structural variables and plasmonic responses, but also in offering important physical insights for experiments and design guidelines for waveguiding plasmonic components and devices. One common approach for modeling MNWs is to utilize FEM or FDTD methods, in which waveguiding properties of these MNWs with different geometry configurations can be numerically obtained. However, the sharp corners and edges in polygonal cross-sections are subject to numerical errors induced by insufficient mesh resolution [13,14,15]. To resolve the abrupt geometric features, one has to resort to an extremely fine mesh with an element size of down to one-to-few or sub-nanometers [14], resulting in extra complexity and a tremendous consumption of computational resources, which cannot meet the urgent demand of large-scale designs in plasmonic circuits and devices. To address this issue, an alternative approach is to approximate the waveguiding properties of polygonal-cross-section MNWs (refer to polygonal MNWs) to circular-cross-section MNWs (refer to circular MNWs) by utilizing the diagonal-circle approximation (DCA) method [16]. In this case, solutions of circular MNWs can be analytically obtained from Maxwell’s equations or numerically obtained with much fewer mesh elements. Despite its inexpensiveness in computational time and resources, such an approach has a relatively low accuracy, leading to large errors in the calculation results (e.g., a deviation of 20% for semiconductor NWs, not to mention MNWs). In fact, as a mixture of plasmons (collective charge oscillations) and optical modes (solutions to Maxwell’s equations at a given geometry), SPPs in MNWs inherit both of their properties and are thus extremely sensitive to the sharp geometric features and the overall symmetry of the system because they greatly modify the surface charge density and influence the hybridization of the optical modes, giving rise to distinct waveguiding properties of polygonal MNWs compared to circular ones.

Besides the aforementioned analytical and numerical methods, deep learning has recently emerged as a powerful data-driven tool for retrieving the photonic/plasmonic properties of nanostructures [17,18,19,20,21,22,23,24,25,26,27,28,29]. However, it is also known to be data hungry, and the task is usually accomplished with a large set of data for training [30,31,32,33], resulting in several challenges. For example, the preparation of the dataset still requires a time- and computational-resource-consuming process [34,35], and the generalization of the trained model of nanostructures with new configurations often has a poor performance [36]. On the other hand, by migrating the learned knowledge from the source task to the related target tasks of a similar problem, transfer learning offers a possible approach to addressing the above challenges [37], and it is especially useful for situations where the target dataset size is limited. Although transfer learning has been successfully utilized in predicting the properties of nanostructures, such as layered nanoparticles [33,38] and metamaterials [39,40], their application in the study of waveguiding properties of MNWs is still an underexploited territory.

To overcome the limitations of conventional deep learnings (e.g., the requirement of large sets of data and an inadequate generalization performance), as well as the drawbacks of non-deep learning methods (e.g., large errors for the approximation approach, and high time and resource consumption for the numerical simulation), we propose a transfer learning approach for modeling MNWs, offering a high performance with a small dataset size and strong generalization capability for various configurations (e.g., MNWs with different cross-sections, working environments, geometric sizes, and wavelengths). The basic idea of this method is to utilize the knowledge acquired in solving the source task of free-standing circular MNWs with source data that is computationally cheap to improve the performance of target tasks for MNWs in complex configurations with small target dataset sizes. We show that, compared to direct learning, our model can achieve a significantly improved performance with much smaller errors (~23–61% relative error reduction) and less trainable parameters (~42% relative size reduction). Moreover, our approach removes the need for a large set of training data, reducing the number of data instances by ~50–80% compared with direct learning. In addition, compared to numerical simulations, our approach enables a much faster computational time, reduced by five orders of magnitude. Meanwhile, compared to other non-deep learning methods, such as the circular-area-equivalence (CAE) approach and the DCA, our approach can offer not only a much higher accuracy, but also a more comprehensive characterization of the waveguiding properties. Benefitting from the advantages of transfer learning, our model provides a simple, lightweight but effective approach to retrieving the plasmonic properties of MNWs with high accuracy, which is much needed in the investigation of plasmonic nano-waveguides. It can greatly accelerate the building of structure–property libraries for plasmonic architectures, revealing hidden relationships between structural variables and plasmonic properties that may open new opportunities to meet the increasing demand for large-scale designs of next-generation nanophotonic circuits and devices.

## 2. Materials and Methods

### 2.1. Emerging Requirement of Computational Resources for MNWs with Sharp Corners and Asymmetric Configuration

**Figure 1:**
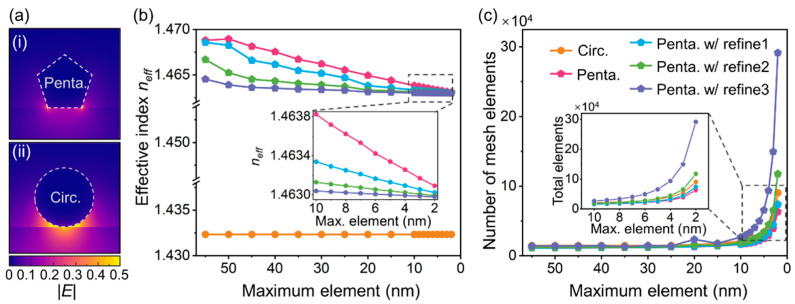
Emerging computational resource requirement for MNWs with abrupt geometric features and broken symmetries. (**a**) Mode profiles in terms of normalized electric field norm distributions in the typical (**i**) pentagonal MNW and (**ii**) circular MNW. For better visualization, they are plotted in a color bar ranging from 0 to 0.5 with saturation. (**b**) Calculated effective index *n_eff_* vs. maximum mesh element size. (**c**) Total number of mesh elements vs. maximum mesh element size of MNWs. Insets in (**b**,**c**): enlarged views for maximum mesh size ranging from 2–10 nm. Orange dotted line: circular MNW. Red, blue, green, and purple lines with pentagonal symbols: pentagonal MNWs with 0–3 times regional meshing refinements (see Figure S1 in the Supplementary Materials for details). The MNW was placed on a silica substrate with a diameter of 300 nm working at 880 nm wavelength.

We first start by demonstrating the emerging requirements of computational resources for MNWs with sharp corners and asymmetric configurations. The numerical simulations were performed with COMSOL Multiphysics (version 6.0, COMSOL AB, Stockholm, Sweden). As a typical case, the silica-substrate-supported pentagonal MNW was selected for demonstration. It can be clearly seen from Figure 1a that the mode profile in the pentagonal MNW was very different from that of circular MNWs, resulting in distinct mode characteristics and waveguiding properties (e.g., pentagonal MNW (bound mode) vs. circular MNW (leaky mode)). Therefore, polygonal MNWs cannot be directly modelled by an approximation to their circular counterparts and must revert to numerical simulations incorporating their sharp features. While in simulations, sharp edges and corners in polygonal MNWs are subject to numerical errors stemmed from meshing, leading to extra complexities and deteriorated accuracies in computation. Taking the calculation of the fundamental waveguiding property—the effective refractive index (*n_eff_*)—as an example (Figure 1b), *n_eff_* of circular MNWs converges at a maximum mesh element size of ~55 nm with a negligible variation in the finer meshing. Meanwhile, for the convergence for pentagonal MNWs, one has to implement an extremely fine meshing with a maximum element size of 4 nm, even with regional refinement techniques (see Figure S1 in the Supplementary Materials for details). As a result of a much smaller meshing size, the total number of mesh elements multiply rapidly (Figure 1c), adding to the consumption of computational time and resources.

### 2.2. Model Architecture with Transfer Learning

In transfer learning, the learned knowledge from one problem can be transferred to multiple problems of the same type, offering opportunities to leverage common features to improve performance and reduce the dataset size [41,42,43]. As for our case, physically, plasmon modes in MNWs were solutions to the source-free Maxwell’s equations in a given configuration (e.g., geometry, wavelengths, and working environments). By defining a time-harmonic electric field ***E*** as ***E***(*x*,*y*,*z*) = ***E***(*x*,*y*)*e^i^*^(*βz*−*ωt*)^, it can be generally described as an eigenvalue problem [44,45]:

(1) $$\begin{array}{l} \mathrm{Find}\;\beta \in \mathbb{C}\;\mathrm{and}\;0\neq E\in H(curl_{\beta },\Omega )\\ s.t.\;curl_{\beta }(\mu_{r}^{-1}curl_{\beta }E)=k_{0}^{2}\varepsilon_{r}E\;\mathrm{in}\;\Omega \end{array}$$

under a boundary condition (e.g., $E\times n|{}_{\partial \Omega }=0$). Here, *ε_r_*, *µ_r_*, and *k*_0_ are the permittivity, permeability, and wavenumber, respectively. *β* is the propagation constant (eigenvalue), Ω is an open set with a boundary ∂Ω, *H* denotes the Hilbert space, and the operator **curl***_β_* is defined as $curl_{\beta }E(x,y)=curl(E\left(x,y\right)e^{i\beta z})e^{-i\beta z}$. Therefore, for MNWs with arbitrary configurations, solving plasmon modes can be regarded as the same type of problem that yields learning transfer among them.

**Figure 2:**
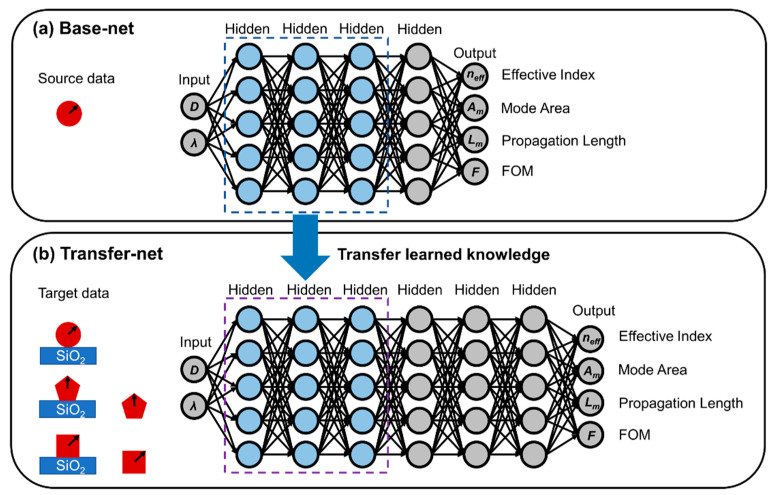
Schematic illustrations of the model architecture. (**a**) Base net and (**b**) transfer net based on ANN frameworks. The base net is used for gaining basic knowledge from free-standing circular MNWs, establishing the mapping from the input of diameters (*D*) and wavelengths (*λ*) to the output of effective index (*n_eff_*), mode area (*A_m_*), propagation length (*L_m_*), and figure of merit (FOM). The transfer net is used to deal with MNWs in complex configurations with abrupt geometric features (free-standing pentagonal and square MNWs) and broken symmetries (silica-substrate-supported circular, pentagonal, and square MNWs). Transfer learning is enabled by migrating learned knowledge (blue neuron nodes within dashed boxes) from the trained base net to the transfer net. The blue rectangles (SiO_2_) represent the silica substrates. The red circles and polygons represent MNWs with different cross-sectional shapes. The diameters *D* of circles/polygons are defined as twice the radii/circumradii (indicated by the black arrows).

By applying the concept of transfer learning, our model was composed of a base net and a transfer net, enabling the migration of the knowledge acquired from a source task (*T_s_*) through the base net to a target task (*T_t_*) with the transfer net. As is schematically shown in Figure 2a, the base net was constructed with a framework of artificial neural networks (ANNs), consisting of the input, output, and 4 hidden layers to learn the physical properties of plasmon modes in free-standing circular MNWs. The associated dataset for training the base net (source data, *D_s_*=$\left\{\left(X_{s},Y_{s}\right)|X_{s}\in {\mathbb{R}}^{m_{s}}{}^{\times 2},Y_{s}\in {\mathbb{R}}^{m_{s}}{}^{\times 4}\right\}$) can be readily obtained at an inexpensive computational cost through numerical simulation because the mesh elements of free-standing circular MNWs are far fewer than those of other configurations. Here, $X_{s}=\{(D^{\left(j\right)},\lambda^{\left(j\right)})|j=(1,m_{s})\}$ represents the 2-dimensional configuration vector (diameter *D*, wavelength *λ*) of MNWs for *m_s_* data instances. Additionally, $Y_{s}=\{(n_{eff}^{\left(j\right)},A_{m}^{\left(j\right)},L_{m}^{\left(j\right)},{\mathrm{FOM}}^{\left(j\right)})|j=(1,m_{s})\}$ represents the corresponding waveguiding properties derived from the physical quantities *β* and ***E***, where *n_eff_*, *A_m_*, *L_m_*, and FOM represent the effective refractive index, propagation length, mode area, and figure of merit, respectively. *n_eff_* and *L_m_* are derived from the real and imaginary part of *β* (Equations (S1) and (S2) in the Supplementary Materials), reflecting the mode characteristics and the spatial decay along the propagation direction (loss), respectively. *A_m_* was calculated from the energy density integration (Equation (S3) in the Supplementary Materials), describing the capability of the energy confinement. In addition, the FOM provides an overall evaluation of the mode quality (Equation (S4) in the Supplementary Materials). A high mode quality indicates a combination of small loss and tight confinement. In this case, the goal of our *T_s_* is to efficiently learn the basic knowledge of the plasmon modes via the learning objective:

(2) $$\min.\sum_{P=1}^{4}\alpha_{P}Loss_{P}(f_{s}(X_{s}),Y_{s})$$

where *f_s_*(⸳) is the source predictive function, *Loss_P_* represents the loss per node in the output layer in terms of mean absolute percentage errors (MAPE), reflecting the difference between the prediction and the actual value, and *α_P_* is the corresponding weight factor. 

On the other hand, for the target task *T_t_* with a predictive learner *f_t_*(⸳), the associated dataset (target data, *D_t_* = $\left\{\left(X_{t},Y_{t}\right)|X_{t}\in {\mathbb{R}}^{m_{t}}{}^{\times 2},Y_{t}\in {\mathbb{R}}^{m_{t}}{}^{\times 4}\right\}$) corresponds to the configuration vector and waveguiding properties of MNWs with broken symmetries or geometric sharp features. The goal of our transfer learning was to simultaneously improve the performance of *f_t_*(⸳) and reduce the number of target data instances *m_t_* via the knowledge acquired from *T_s_*. To achieve this, a transfer net incorporating 6 hidden layers was deployed, in which the first 3 hidden layers with fixed weights and biases (transferred layers) were transferred from the base net containing the general features extracted in solving *T_s_*, and the rest of the hidden layers were designed to learn the new features of the plasmon modes (Figure 2b). It is worth noting that such a design of the transfer net was also under the guidance of physics. From the physical point of view, the symmetry breaking or geometric sharpness actually lifts the degeneracy of the MNW system, leading to the hybridized mode generated by the coupling between the original symmetric modes [2]. Therefore, the hybridized plasmon mode contains, but is not limited to, the features of the symmetric mode extracted from the circular MNWs.

The TensorFlow framework was used to construct the ANN. To train our model, the dataset we used contained 1680 groups of data obtained from the numerical simulation of MNWs with 6 types of configuration (280 groups for each), including free-standing and substrate-supported MNWs with circular, square, and pentagonal cross-sections. For each MNW configuration, the dataset was divided into the training dataset (~60%), the validation dataset (~20%), and the test dataset (~20%). Typical geometric sizes and operation wavelengths were considered to cover broad ranges of *D* (40–300 nm) and *λ* (520–900 nm) in the visible and near-infrared bands. To minimize the loss function in the training process, the Adam optimizer with an initial learning rate of 10^−3^ and a decaying rate of 0.99 was applied. The base net was firstly trained, and after training the base net, the transferred layers with fixed trainable parameters were transferred to the transfer net. The trainable parameters for the rest of the hidden layers in the transfer net were then initialized by random normal initialization for the training of the transfer net. For performance evaluation, due to the values of different waveguiding properties across several orders of magnitude, the individual performance of each of the four properties (*n_eff_*, *A_m_*, *L_m_*, FOM) was evaluated from its MAPE on a given property *P*:

(3) $$\sigma_{P}=\frac{100\%}{m_{test}}\sum_{i=1}^{m_{t}{}_{est}}\left|\frac{{\hat{P}}_{i}-P_{i}}{P_{i}}\right|$$

where $\hat{P_{i}}$ and *P_i_* represent the predicted and actual property, and *m_test_* is the number of data instances in the test set. We also calculated the average of the four MAPEs ($\sum {\mathrm{MAPE}}_{P}/4$) to assess the average error *σ_avg_* of our model.

## 3. Results and Discussion

### 3.1. Optimized Layout for Gaining the Basic Knowledge

**Figure 3:**
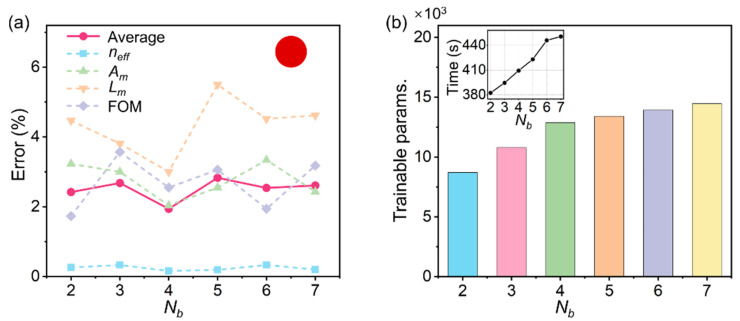
Optimized layout for the base net. (**a**) Dependence of errors on the number of hidden layers (*N_b_*) in the base net. Dashed lines with squares, up triangles, down triangles, and diamonds represent the individual error of effective index (*n_eff_*), mode area (*A_m_*), propagation length (*L_m_*), and figure of merit (FOM), respectively. Red solid line with dots: overall performance in terms of the average error. Inset: schematic illustration of the geometry of the circular MNW. (**b**) Number of trainable parameters vs. *N_b_*. Inset: an increasing training time with the increase in *N_b_*.

We first discuss the optimized layout for the base net, which was used to gain basic knowledge of the plasmon modes by feeding a large set of training data *D_s_*. Therefore, it is desirable to be lightweight and to have a small number of trainable parameters and an efficient training time without sacrificing the performance of *f_s_*(⸳). For this purpose, the number of hidden layers (*N_b_*) in the base net was determined by an overall evaluation of errors, trainable parameters, and training time. As is shown in Figure 3, the base net with four hidden layers was able to achieve the overall optimized performance, offering a minimum average error *σ_avg_* of 1.94% (the corresponding individual errors *σ_neff_*, *σ_Am_*, *σ_Lm_*, and *σ*_FOM_ are 0.16%, 2.04%, 3.00%, and 2.55%, respectively) compared to all other choices of *N_b_*. Note that, although the base net with six hidden layers (*N_b_* = 6) can achieve a smaller error of the FOM (1.94%) compared to the *N_b_* = 4 case, it has a higher average error (2.54%) with more parameters that need to be trained (Figure 3b) and, consequently, a longer time needed for training (Figure 3b inset).

### 3.2. Performance Improvement in f_t_(⸳) and Reduction in Training Parameters

After training the base net with the optimized layout, a certain number of hidden layers (*N_t_*) in the base net containing the learned knowledge were copied to the transfer net to deal with MNWs of complex configurations. For comparison with the conventional direct learning (DL) approach without the learning transfer, the performance improvement in *f_t_*(⸳) was evaluated by the change in the average error as:

(4) $$\Delta (N_{t})=100\%\frac{\sigma_{avg}(0)-\sigma_{avg}(N_{t})}{\sigma_{avg}(0)}$$

where *σ_avg_*(*N_t_*) is the average error of the transfer net when *N_t_* layers are transferred, and *σ_avg_*(0) represents the corresponding error via DL (*N_t_* = 0, initializing and training all parameters in the transfer net). Under the above definition, Δ(*N_t_*) > 0 indicates a positive transfer, such that the knowledge stored in the transferred layers facilitates the learning of *f_t_*(⸳). Meanwhile a negative Δ(*N_t_*) indicates the opposite situation, and is known as a negative transfer. Meanwhile, the larger the Δ(*N_t_*), the greater the performance improvement.

**Figure 4:**
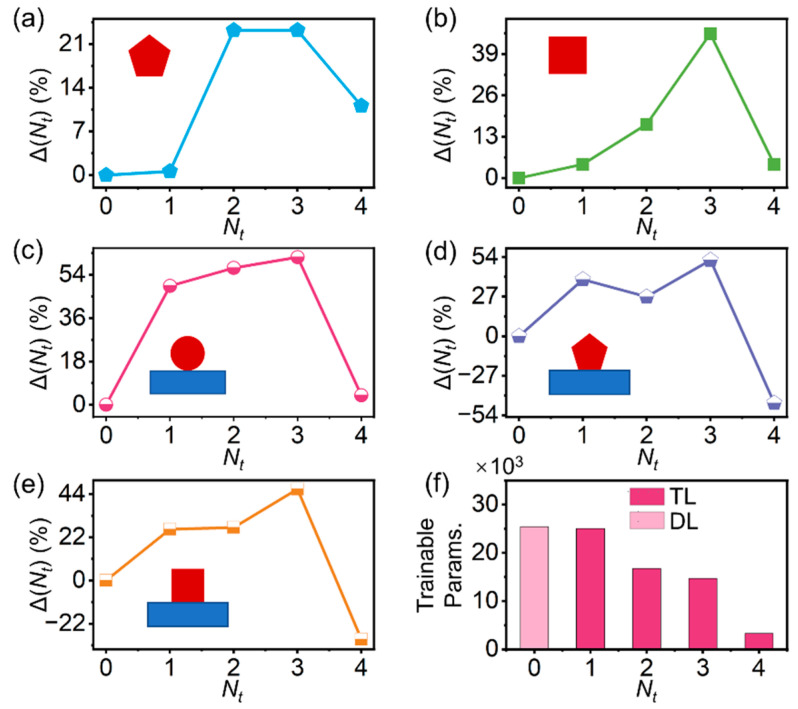
Significantly improved performance with reduced trainable parameters enabled by transfer learning. Dependence of the performance improvement on the number of transferred layers (*N_t_*). (**a**) free-standing pentagonal MNWs, (**b**) free-standing square MNWs, (**c**) substrate-supported circular MNWs, (**d**) substrate-supported pentagonal MNWs, and (**e**) substrate-supported square MNWs. (**f**) Comparison of trainable parameters between direct learning (DL) and the transfer learning (TL). Insets: schematic illustrations of the geometries of MNWs with different configurations.

To demonstrate the effectiveness and generality of our model, typical scenarios of MNWs with geometric abruptness or/and broken symmetries, including free-standing pentagonal MNWs (fp-MNWs), free-standing square MNWs (fs-MNWs), substrate-supported circular MNWs (sc-MNWs), substrate-supported pentagonal (sp-MNWs), and substrate-supported square MNWs (ss-MNWs) were investigated. As is shown in Figure 4, Δ(*N_t_*) increases with more layers being transferred until reaching their minimum at *N_t_* = 3, exhibiting an exceptional performance that is superior to the DL and generalization capability that is applicable for the MNW with every new configuration. For all cases at *N_t_* = 3, the performance improvements of fp-, fs-, sc-, sp-, and ss-MNWs were 23.2%, 45.4%, 61.3%, 51.9%, and 46.5% compared to DL, yielding excellent average errors (*σ_avg_*(3)) as small as 2.48%, 1.65%, 2.60%, 1.75%, and 2.60%, respectively. Compared to the improvements with learning transfers that were demonstrated in other nanostructures (e.g., among multi-layered films (~23–50%), from multi-layered nanoparticles to multi-layered films (~20%) [33]), a greater enhancement can be achieved for our MNWs, providing an effective way to migrate knowledge across varied configurations in the waveguiding system. It is also worth mentioning that, besides the performance improvement, transfer learning also enabled a reduced number of trainable parameters compared to DL (e.g., ~42% relative reduction in our model, Figure 4f), with a more efficient training process for every new task, consequently, because the transfer layers embedded in the transfer net were already pre-trained. 

On the other hand, when we transferred all the hidden layers from the trained base net (*N_t_* = 4), Δ(*N_t_*) dramatically decreased, resulting in a deteriorated performance and even negative transfers for the sp-MNWs and ss-MNWs. Such behaviors correspond well to the characteristics of ANNs. Generally, the features on each layer of the ANN evolve from general to specific along with the network, and the last layer is therefore very specific to a particular problem [46]. In our base-net case, the specialization of the fourth layer of the circular free-standing MNW for *T_s_* made it inapplicable for *T_t_*, and the transfer of such a layer will only lead to deterioration, rather than the enhancement of the performance for modeling MNWs with other new configurations.

### 3.3. Removing the Need for a Large Set of Training Data with Reduced m_t_

**Figure 5:**
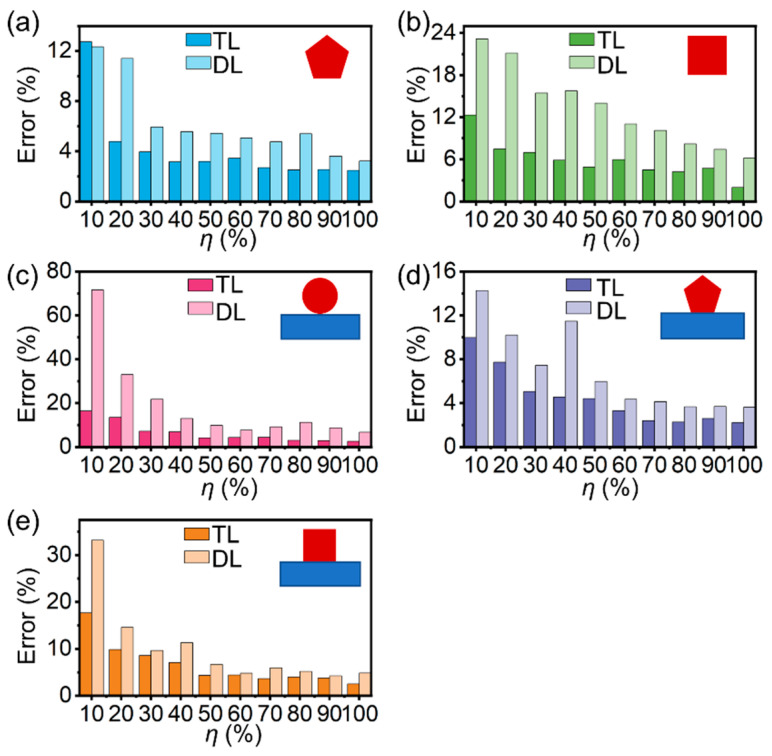
Reduced training dataset size enabled by transfer learning. Comparison of average errors between transfer learning and direct learning using different portions (*η*) of the training dataset for (**a**) free-stranding pentagonal MNWs, (**b**) free-standing square MNWs, (**c**) substrate-supported circular MNWs, (**d**) substrate-supported pentagonal MNWs, and (**e**) substrate-supported square MNWs. Insets: schematic illustrations of the geometries of MNWs with different configurations.

In addition to the performance improvement with the reduction in trainable parameters, our transfer learning model also removes the requirement for a large set of training data *D_t_* for MNWs with every new configuration. To demonstrate, using only a small portion of the training dataset (*η* = 100%*m_t_*/*m_tot_*, where *m_t_* is the number of data instances used for training and *m_tot_* is the total number of data instances in the training dataset), we evaluated the *η*-dependent *σ_avg_* for fp-, fs-, sc-, sp-, and ss-MNWs (Figure 5), and the corresponding performance of direct learning is also provided for comparison. As is shown, for a given *η*, the performance of transfer learning was much better than direct learning. Additionally, for a given *σ_avg_*, transfer learning enables successful training with a much smaller *η*. For example, to maintain an acceptable *σ_avg_* of ~5%, only 20% (*m_t_* = 33), 50% (*m_t_* = 83), 50% (*m_t_* = 83), 40% (*m_t_* = 66), and 50% (*m_t_* = 83) portions of training datasets are required for fp-, fs-, sc-, sp-, and ss-MNWs, respectively. Such an ability in achieving high performance with small datasets is able to greatly reduce the time of not only training, but also data preparation, that is computationally expensive through numerical simulations, significantly accelerating and facilitating the training and data acquisition process.

### 3.4. Accurate, Effective and Comprehensive Mapping of Waveguiding Properties 

With the merits of excellent performance and reduced dataset size, our transfer learning model circumvents the drawbacks of conventional deep learning, providing an accurate and efficient way to model MNWs with a high generalization capability. Moreover, our model also exhibits an overwhelming performance compared to non-deep learning methods (e.g., DCA and CAE [16]). For comparison, *σ_neff_* calculated by our model was 0.33%, 0.18%, 0.12%, 0.23%, and 0.20% for the fp-, fs-, sc-, sp-, and ss-MNWs, which is one order of magnitude smaller than the ones of the DCA and CAE methods (~7% of DCA method and ~8% for the CAE method, see Figure S2 in the Supplementary Materials for details). Such a huge improvement is crucial for various situations where an accurate propagation constant is required (e.g., routers, couplers, and correlators) [47]. Besides the accurate prediction of *n_eff_*, our model is able to obtain other waveguiding properties (*L_m_*, *A_m_*, and FOM) that have not been demonstrated by the DCA and CAE methods, yielding a comprehensive study of the plasmon modes in MNWs. 

**Figure 6:**
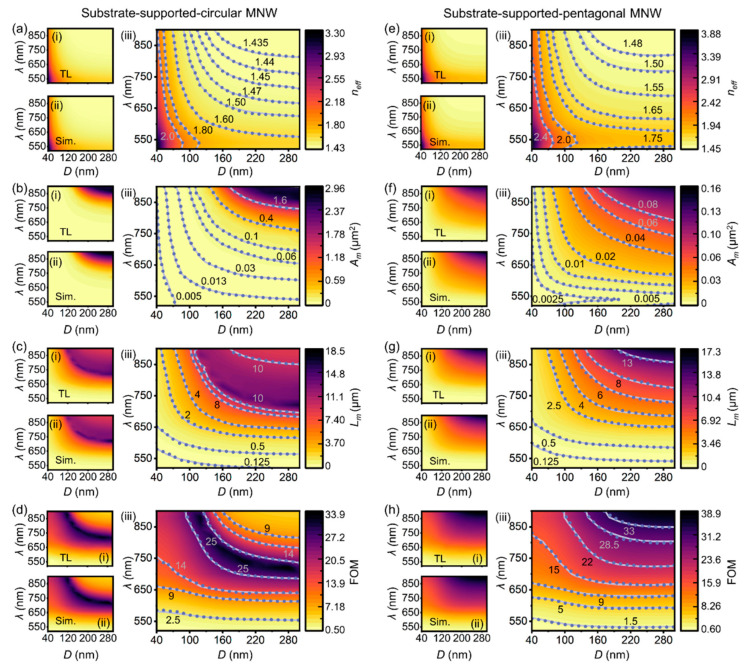
Accurate, effective, and comprehensive mappings of waveguiding properties of MNWs with different configurations enabled by our model. Waveguiding properties of (**a**–**d**) substrate-supported circular MNWs and (**e**–**h**) substrate-supported pentagonal MNWs over a broad range of diameters (*D*) and wavelengths (*λ*). (**a**,**e**) Effective index (*n_eff_*), (**b**,**f**) mode area (*A_m_*), (**c**,**g**) propagation length (*L_m_*), (**d**,**h**) figure of merit (FOM). Predictions by our model (TL, (**i**) in (**a**–**h**)) coincide well with the numerical simulations (Sim., (**ii**) in (**a–h**)), which are further verified by overlaying the images of the TL and Sim. results ((**iii**) in (**a**–**h**)). For reference, contour lines are also provided in (**iii**), where the TL (purple dotted lines) exhibit excellent agreement with the Sim. (light purple solid lines).

As an illustration, Figure 6 gives *λ*-*D* mappings of *n_eff_*, *A_m_*, *L_m_*, and FOM for sc-MNWs (Figure 6a–d) and sp-MNWs (Figure 6e–h) using our model (see all configurations in Figure S3, Supplementary Materials). They exhibit no visual discrepancy over the broad ranges of *λ* and *D* compared to the results from numerical simulations (TL in Figure 6(ai)–(hi) vs. Sim. in Figure 6(aii)–(hii)). We further overlayed the results from the simulation and our model and provided the contour lines for better visualization (Figure 6(aiii)–(hiii)). As shown, the results obtained from our model (purple dotted lines) coincides very well with the ones using numerical simulations (light purple solid lines), while the time consumptions, by contrast, were reduced by five orders of magnitude (~560 ms for TL vs. ~12 h for Sim.). Therefore, our model offers an effective and effortless approach to systematically characterize the plasmonic waveguiding properties with varied configurations. Taking the mode characteristics as an example, with the increasing *λ* and *D*, the plasmon mode in the sc-MNW transits from the bound mode (*n_eff_* > 1.45) to the leaky mode (*n_eff_* < 1.45) in the region above the transition line (purple line with the label 1.45 in Figure 6(aiii)), while the plasmon mode in the sp-MNW is always the bound mode within the range of *λ* and *D* presented. Therefore, even at the same *λ* and *D*, the pentagonal and circular MNWs exhibit distinct *A_m_* and *L_m_*, with differences that can be as large as one order of magnitude (e.g., Figure 6b vs. Figure 6f), resulting in totally different application scenarios. In addition to revealing the mode characteristics, the generated plasmonic mappings also facilitate the optimization of the trade-off relation between confinement and loss, which lies at the heart of the design of plasmonic components and devices. The optimized trade-off can be achieved when the FOM reaches its maximum value. As shown in the FOM mappings generated by our model (Figure 6d,h), the local maximum FOM at a desirable *D* or *λ* can be found, as well as the global maximum FOM for all the *D* and *λ* combinations can be revealed. This result also indicates the ability to uncover relationships between the functional properties and design variables (e.g., cross-sectional shapes, working environments, geometric sizes, and wavelengths), which can offer a valuable reference and new opportunities for designing high-performance plasmonic components and devices.

## 4. Conclusions

In summary, based on a transfer learning approach, we have proposed a general model for predicting the waveguiding properties of MNWs of arbitrary cross-sectional shapes and working environments with varied geometric sizes and wavelengths. The model consists of a base net for learning the basic knowledge from the simple case of free-standing circular MNWs, and a transfer net for dealing with complex MNW configurations. The dependence of errors on the base-net layers and the transferred layers have been investigated to achieve the optimized performance. In addition, the conditions for the positive transfer and the negative transfer have been analyzed to give an insight into our neural network structure. We have showed that, by migrating the learned knowledge from the source task of the base net to the target tasks of the transfer net, the performance of the target tasks can be greatly improved, enabling much smaller errors with less trainable parameters than direct learning. Meanwhile, our model also works well with small datasets, saving ~50–80% of the number of data instances than direct learning, which greatly reduces the time of data preparation through numerical simulation. The generality and robustness of this approach has also been demonstrated by accurate predictions of various MNW configurations with broken symmetries or/and different cross-sectional shapes. Moreover, we have also demonstrated that, compared to other methods (DCA and CAE) that are only capable of retrieving the effective index, our approach enables not only a much higher accuracy over a broad range of diameters and wavelengths, but also a more comprehensive characterization of the waveguiding properties, reflecting the trade-off between confinement and loss. Additionally, compared to numerical simulations, time consumption is reduced by five orders of magnitude. Benefitting from advantages, including a high performance with generalization capabilities, simple architecture with a small-scale neural network, and a lightweight dataset with a reduced size, our approach offers an effective route for accurately retrieving the plasmonic properties of MNWs without extensive training time and data, which may greatly facilitate the investigation and design of plasmonic/polaritonic components and devices.

## Data Availability

The data presented in this study are available on request from the corresponding author.

## Supplementary Materials

The following supporting information can be downloaded at: https://www.mdpi.com/article/10.3390/nano12203624/s1, Figure S1: Illustration of the refinement method.; Figure S2: Mappings of waveguiding properties of MNWs with different configurations.; Figure S3: Dependences of effective indices and errors on normalized diameters. References [48,49,50,51] are cited in the supplementary materials.
