# Supplementary material for: Transfer Learning for Modeling Plasmonic Nanowire Waveguides
Source: Nanomaterials (Basel). 2022 Oct 16;12(20):3624. doi: 10.3390/nano12203624 (PMC9612048; doi:10.3390/nano12203624)
Supplement: Supplementary file 1 [file nanomaterials-12-03624-s001.zip › nanomaterials-1969179-supplementary.pdf]

nanomaterials

Article

Transfer Learning for Modeling Plasmonic Nanowire Waveguides

Aoning Luo, Yuanjia Feng, Chunyan Zhu, Yipei Wang * and Xiaoqin Wu *

Key Laboratory of Optoelectronic Technology and Systems (Ministry of Education), College of Optoelectronic Engineering, Chongqing University, Chongqing 400044, China
* Correspondence: wangyp@cqu.edu.cn (Y.W.); xiaoqinwu@cqu.edu.cn (X.W.)

1. Numerical simulations with regional refinement techniques

For numerical simulations with COMSOL Multiphysics, the computational domains, terminated by perfectly matched layer boundaries, are meshed with triangular meshing elements. For the domain of the dielectric environment, we set the maximum size of the meshing element to be $\lambda/12$. While for the metal nanowire, we first set a maximum element size (size_max) for the initial mesh, and then refine the mesh m times (m=1~3) in the regions containing sharp geometric features by using the regular refinement method. For each time of the refinement, a triangular element is divided into 4 small triangular elements by bisecting all edges of the original element, as shown in Figure S1. The value of maximum element size (size_max) and refinement numbers (m) are determined according to the convergence tests until the calculated parameters remain unchanged. Au with the permittivity taken from Ref. [1] is selected as the nanowire material. For the free-standing case, the surrounding is set to be air with a refractive index of 1. For the substrate case, a typical silica substrate (SiO_2) with a refractive index of 1.45 is used for simulation. For calculation, we only focus on the fundamental TM_0 mode since it dominates the waveguiding properties and the single-mode operation is favorable which can be readily realized in practical applications.

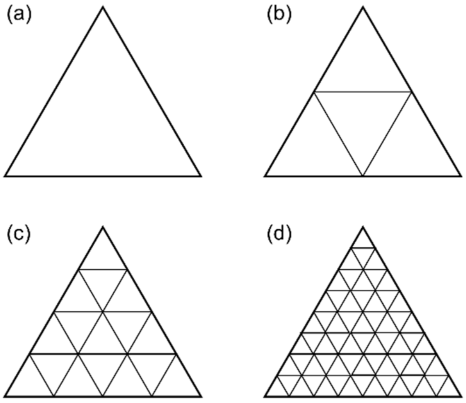

Figure S1. Illustration of the refinement method. (**a**) m=0 without refinement, (**b**) m=1, (**c**) m=2, and (**d**) m=3.

2. Equations for calculating waveguiding properties

The effective index (n_{eff}) is defined as

$$n_{eff} = \frac{|\mathrm{Re}(\beta)|}{k_0}, \tag{S1}$$

where β is the propagation constant, and k_0 is the free-space wavevector. The propagation length is calculated as [2]

$$L_m = \frac{1}{2|\mathrm{Im}(\beta)|}. \tag{S2}$$

The effective mode area (A_m) is calculated by [3]

$$A_m = \frac{[\int W(r)dA]^2}{\int W(r)^2 dA}, \tag{S3}$$

where $W(r)$ is the energy density, the integration area is over the whole mode, and dA denotes the cross-sectional surface element. Note that, the definition of A_m here represents a statistical measure, which will be larger than the values calculated by the ratio definition (the total energy density of a mode divided by its peak energy density). Compared to the mode area calculated by the ratio definition that is greatly affected by the sharp geometric features with inconsistent portions of energy included, the statistical measure A_m is able to provide a consistent measure of the total mode energy [3], allowing a fairer comparison between MNWs with different configurations in our case. The figure-of-merit (FOM) is used to evaluate the performance of the waveguiding mode, which can be expressed as [4]

$$\mathrm{FOM} = \frac{L_m}{2\sqrt{A_m/\pi}}. \tag{S4}$$

As can be seen from Eq. S4, larger FOMs denote longer propagation lengths with smaller mode areas, indicating waveguiding modes with better performance.

3. Technical details about the ANN

For more details, the open-source TensorFlow framework is used to construct the ANN. The optimized model for the base-net has 4 hidden layers with 128, 64, 32, 16 per hidden layer. And the optimized model for the transfer-net has 6 hidden layers with 128, 64, 32, 128, 64, and 32 neurons per hidden layer. The nonlinear ReLU function is selected as the activation function for neurons. The loss function representing the total loss of the nodes in the output layer is defined as their weighted linear combination of the mean absolute percentage errors (MAPE) reflecting the difference between the prediction and the actual value of numerical simulation. The weight factors in the loss function are empirically obtained based on the convergence to scale the loss of each output node and emphasize the loss of n_{eff} due to its fundamental importance governing the waveguiding properties. Due to the range of the data for different waveguiding properties across several orders of magnitude, MinMax normalization is used to scale the data to the range of [0,1]. Since the normalized data points with zero values will generate infinite MAPEs, they are treated as outliers and excluded in the training dataset. To minimize the loss function, Adam optimizer with initial learning rate of 10^{-3} and decaying rate of 0.99 are applied. After training the base-net, the first 3 hidden layers with fixed trainable parameters are transferred to the transfer-net. The trainable parameters for the rest of the hidden layers and the output layer in the transfer-net are then initialized with random normal initialization for the training.

4. Performance of circular-area-equivalence approach (CAE) and diagonal-circle approximation (DCA).

To evaluate the performance of CAE and DCA, Figure S2 gives dependences of effective indices (n_{eff}) and errors on normalized diameters (D/λ).

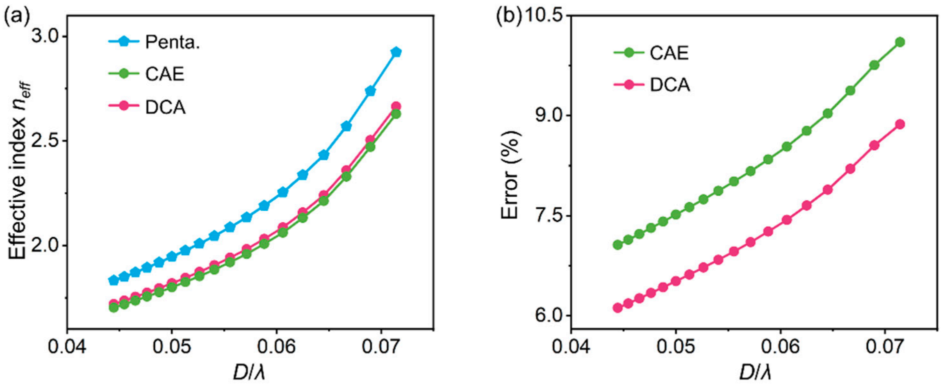

Figure S2. Dependences of effective indices and errors on normalized diameters. (**a**) Calculated effective indices and (**b**) errors vs. D/λ. Blue line with pentagonal symbols: numerical simulation results for free-standing-pentagonal MNWs. Green and red lines with circular symbols: results using CAE and DCA. The diameter of the MNW is 40 nm.

5. Mapping of waveguiding properties for MNWs

Beside the cases of substrate-supported-pentagonal and -circular MNWs shown in the main text, Figure S3 gives mappings of MNWs with other configurations. All of them exhibit excellent agreements to the numerical simulations.

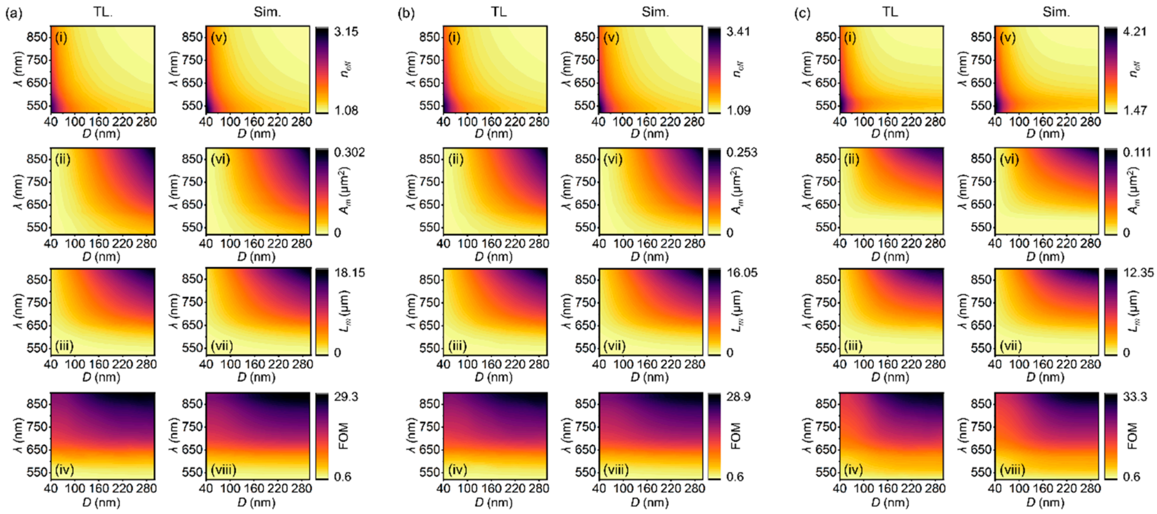

Figure S3. Mappings of waveguiding properties of MNWs with different configurations. (**a**) free-standing-pentagonal MNWs, (**b**) free-standing-square MNWs, and (**c**) substrate-supported-square MNWs over a broad range of diameters (D) and wavelengths (λ). Predictions by our model (TL) of (i) effective index (n_{eff}), (ii) mode area (A_m), (iii) propagation length (L_m), and (iv) figure-of-merit (FOM), show excellent agreement with the numerical simulations (Sim., v-viii).

References

1. Johnson, P.B.; Christy, R.W. Optical Constants of the Noble Metals. *Phys. Rev. B* **1972**, *6*, 4370–4379.
2. Maier, S.A. *Plasmonics: Fundamentals and Applications*; Springer, New York, NY, USA, 2007.
3. Oulton, R.F.; Bartal, G.; Pile, D.F.P.; Zhang, X. Confinement and propagation characteristics of subwavelength plasmonic modes. *New J. Phys.* **2008**, *10*, 105018.
4. Zhang, S.; Xu, H. Optimizing Substrate-Mediated Plasmon Coupling toward High-Performance Plasmonic Nanowire Waveguides. *ACS Nano* **2012**, *6*, 8128–8135.
